# Stem design in radial head arthroplasty: a systematic review and meta-analysis

**DOI:** 10.1016/j.jsea.2026.100052

**Published:** 2026-06-26

**Authors:** Harry Beale, Olivia O'Malley, Chuck Lam, Neil Jones, Peter Reilly, Angela Kedgley, Sanjeeve Sabharwal

**Affiliations:** a Imperial College London, London, UK; b Imperial College Healthcare NHS Trust, London, UK; c Barts Health - Royal London, London, UK

**Keywords:** Radial head arthroplasty, Elbow, Complications, Revision, Prognostic factors, Fixation, Implant polarity, Meta-analysis

## Abstract

**Background:**

Radial head arthroplasty (RHA) is increasingly used for complex elbow instability, yet controversy persists over optimal stem fixation and implant polarity. This study systematically reviews the evidence to determine revision rates, radiographic loosening, and reoperation rates after primary RHA, stratified by stem fixation (intentional loose-fit, press-fit, cemented) and polarity (monopolar vs. bipolar), with specific attention to surveillance time bias.

**Methods:**

MEDLINE, Embase, Scopus, and Cochrane databases were searched from inception to October 20, 2025. Studies reporting outcomes of primary RHA with ≥12 months of follow-up and design-specific data were included. Risk of bias was assessed with the Methodological Index for Non-Randomized Studies tool and quality of evidence using Grading of Recommendations Assessment, Development and Evaluation. Both conventional pooled-proportion meta-analysis and incidence-rate (events per 100 patient-years) meta-analysis were performed to adjust for differences in follow-up duration.

**Results:**

Forty-six studies (4,467 distinct elbows) were included. In the unadjusted pooled analysis, intentional loose-fit stems demonstrated a lower revision rate (3.4% vs. 8.0%; *P* < .001) compared to press-fit stems. However, after adjusting for exposure time in the incidence-rate meta-analysis, this survival advantage was no longer significant, although the point estimate for press-fit stems remained higher (loose-fit 0.97 vs. press-fit 2.01 revisions/100 patient-years; *P* = .10). No difference was found between monopolar and bipolar designs in any analysis (revision 5.8% vs. 5.8%; *P* = .74).

**Conclusion:**

This large RHA meta-analysis shows the widely reported survival advantage of intentional loose-fit stems may be partly attributable to shorter follow-up in those cohorts. After adjustment for surveillance time, the difference in revision rates between loose-fit and press-fit stems was no longer statistically significant, although the point estimate for press-fit stems remained numerically higher. Implant polarity was not associated with revision risk in any analysis. The additional complexity and cost of bipolar implants may not translate into improved revision-free survival in this dataset.

Radial head arthroplasty (RHA) is the standard treatment for unreconstructable radial head fractures associated with instability.^26^^,^^51^^,^^67^ As indications expand to include younger, higher-demand patients, the mechanical longevity of these implants has become critical.^14^ Despite the procedure’s prevalence, optimal prosthetic design remains debated, with contemporary implants following 2 opposing philosophies: stem fixation and head-neck polarity.

Fixation strategies diverge fundamentally on the role of osseointegration. Press-fit stems rely on textured surfaces for rigid biological fixation, requiring precise sizing to prevent loosening.^1^ In contrast, intentional loose-fit designs use smooth stems that act as intramedullary spacers; this allows for controlled micromotion and toggling to accommodate minor malalignments.^28^ A secondary controversy surrounds implant polarity. Bipolar designs incorporate a mobile polyethylene bearing intended to optimize radiocapitellar tracking and minimize erosive wear on the native capitellum.^43^ In contrast, monopolar designs offer a monolithic construct that eliminates failure modes associated with modularity, such as polyethylene wear or dissociation,^66^ although concerns persist regarding potential point-loading and capitellar erosion.^12^

While recent systematic reviews have favored intentional loose-fit stems,^1^^,^^2^^,^^67^ the literature remains contested.^28^^,^^32^^,^^63^ Furthermore, these comparisons are likely confounded by surveillance bias. Cohorts evaluating press-fit stems frequently report longer follow-up durations than those for loose-fit designs, naturally accumulating higher raw revision counts over the extended observation period.^14^ Standard meta-analyses that fail to adjust for this disparity in exposure time may unfairly penalize designs with longer surveillance. This study uses incidence-rate meta-analysis to normalize failure events per 100 patient-years, determining if the perceived advantage of loose-fit stems is a mechanical reality or a statistical artifact.

## Methods

### Search strategy and eligibility

This systematic review and meta-analysis was conducted and reported in accordance with the Preferred Reporting Items for Systematic Reviews and Meta-Analyses guidelines.^48^ The protocol was prospectively registered on International Prospective Register of Systematic Reviews (CRD420251155561).

MEDLINE, Embase, and Scopus were searched from database inception to October 20, 2025. The search combined Medical Subject Headings terms and free-text words for “radial head” with terms for arthroplasty, replacement, or prosthesis, unrestricted by outcome (full strategy in Supplementary Fig. 1). Bibliographies of included studies and prior reviews were hand-searched.

Studies were included if they reported outcomes of primary RHA in adults (aged >18 years) with a minimum mean or median follow-up of 12 months and provided data stratified by stem fixation philosophy (intentional loose-fit, press-fit, or cemented) or implant polarity (monopolar vs. bipolar). Larger registry reports were excluded unless they provided implant-specific stratification sufficient to attribute events accurately to design.

Intentional loose-fit stems were defined as smooth, highly polished implants intentionally designed to function as intramedullary spacers with controlled micromotion and no reliance on osseointegration for stability.^26^^,^^30^^,^^66^ Press-fit stems were defined as those with textured, grit-blasted, plasma-sprayed, or porous-coated surfaces intended for rigid biological fixation.^1^^,^^28^^,^^67^ Cemented stems formed a separate category.

Studies reporting exclusively on silicone implants or revision arthroplasty were excluded. Non-English language publications, conference abstracts, and series with less than 20 cases were also excluded.

Two reviewers (H.B. and N.J.) independently screened titles, abstracts, and full texts. Disagreements were resolved by a third reviewer (O.O.).

### Data extraction and quality assessment

Data were extracted in duplicate using a piloted form and included patient demographics, indication (acute trauma vs. sequelae or arthritis), implant brand, polarity, fixation type, follow-up duration, revision, and reoperations. The primary outcome was revision (removal or exchange of any prosthetic component). Secondary outcomes were reoperations (any unplanned elbow procedure not meeting the definition of revision). Risk of bias was assessed using the Methodological Index for Non-Randomized Studies tool.^58^ Evidence quality was graded using the Grading of Recommendations Assessment, Development and Evaluation framework.^24^

### Statistical analysis

Statistical analysis was performed using R version 4.4.0 (R Foundation for Statistical Computing, Vienna, Austria) with the meta package.^6^

Pooled proportions were estimated using random-effects generalized linear mixed models with logit transformation^60^; for outcomes with low or zero events, the Freeman-Tukey double-arcsine transformation was applied. Heterogeneity was assessed using the I^2^ statistic^29^ and Tau.^2^

To control for surveillance bias,^35^ an incidence rate meta-analysis was performed. Revision rates were calculated as an incidence density (events per 100 patient-years) to adjust for variations in exposure time.^60^ The total exposure time (patient-years at risk) was derived by multiplying the number of elbows in each study cohort by the reported mean duration of follow-up. Follow-up duration is typically right-skewed; however, given the absence of individual patient data and to maximize study inclusion, Wan et al’s method was used to estimate the mean and standard deviation from the median and range, as per prior orthopedic meta-analyses.^68^

Incidence rate ratios (IRRs) were compared between groups to determine statistical significance independent of surveillance time. Subgroup analyses were performed to compare outcomes by fixation strategy and implant polarity. To reduce type I error inflation from multiple comparisons, a significance level of 0.01 was chosen a priori.

To explore sources of between-study heterogeneity, random-effects meta-regression was performed on the log incidence rate at the subgroup level, using the DerSimonian-Laird estimator for between-study variance (τ^2^). A continuity correction of 0.5 was applied to zero-event subgroups. Prespecified moderators were stem fixation philosophy (loose-fit, press-fit, cemented), study-level mean patient age, study-level mean follow-up duration, and publication year. Univariable models were fitted for each moderator and a multivariable model combining fixation, mean age, and publication year was used to assess independent effects. Residual I^2^ was reported as an indicator of the proportion of heterogeneity unexplained by each model.

Institutional review board approval was not required for this systematic review and meta-analysis, as it involved only the secondary analysis of previously published, aggregate data and did not access individual patient-level information.

## Results

### Study characteristics

**Figure 1:**
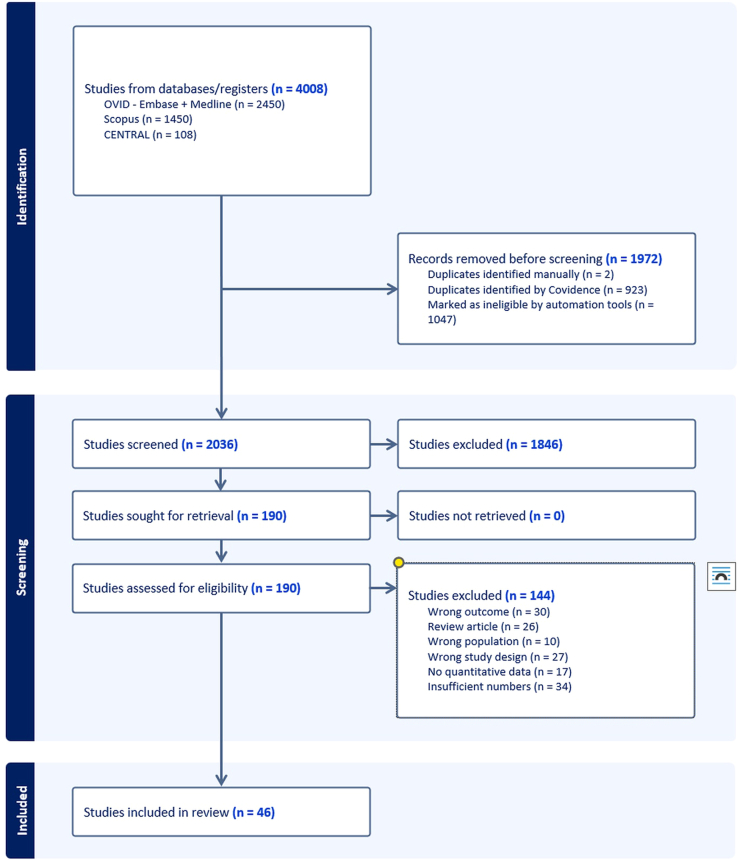
PRISMA 2020 flow diagram detailing the study selection process. From an initial identification of 4,008 records, 46 studies met the inclusion criteria for the final meta-analysis. *PRISMA*, Preferred Reporting Items for Systematic Reviews and Meta-Analyses.

The updated searches to October 20, 2025 identified 4,008 records. Following the removal of duplicates and screening, a total of 46 studies comprising 4,467 primary RHAs were included in the final analysis (Fig. 1; Table I). Median follow-up was 49 months (range: 12-204 months). Thirty-one studies (70%) were retrospective case series, 11 (25%) prospective cohort studies, and 2 randomized trials. The majority (82%) were level IV evidence.

**Table I:** Characteristics of the 46 included studies and 4,467 primary radial head arthroplasties

Author	Mean age (yrs)	Mean follow-up (yrs)	N	Implantation	Polarity	Events (revisions)
Allavena et al^3^ (2014)	53	4.2	22	Cemented	Bipolar	4
Baek et al^5^ (2020)	48	4.9	24	Press-fit	Monopolar	0
Bindal et al^7^ (2025)	38	0.5	26	Press-fit	Monopolar	0
Campbell et al^8^ (2025)	54	8.1	89	Mixed	Monopolar	4
Celli et al^9^ (2024)	53	12.4	23	Cemented	Bipolar	3
Chen et al^11^ (2021)	52	10.0	24	Press-fit	Monopolar	0
Ciais et al^13^ (2025)	59	5.9	139	Press-fit	Monopolar	13
Díez-Sánchez et al^15^ (2023)	54	5.1	95	Mixed	Mixed	9
Dillon et al^16^ (2022)	51	3.8	312	Mixed	Mixed	35
Duckworth et al^17^ (2014)	51	6.7	105	Press-fit	Monopolar	3
Eyre-Brook et al^19^ (2025)	55	5.7	96	Press-fit	Bipolar	7
Flinkkila et al^20^ (2012)	48	4.2	42	Press-fit	Monopolar	9
Galavotti et al^21^	55	2.6	124	Press-fit	Bipolar	13
Gramlich et al^22^ (2019)	54	3.5	66	Press-fit	Monopolar	7
Grewal et al^23^ (2006)	49	2.0	26	Loose-fit	Monopolar	0
Hari Krishnan et al^25^ (2019)	42	1.5	30	Press-fit	Monopolar	0
Harrington et al^1^ (2001)	45	12.1	20	Loose-fit	Monopolar	0
Heijink et al^27^ (2016)	56	4.2	25	Cemented	Bipolar	1
Kachooei et al^31^ (2016)	49	3.5	278	Mixed	Mixed	22
Klug et al^33^ (2023)	51	4.1	65	Mixed	Monopolar	20
Kooi et al^34^ (2025)	52	1.0	128	Loose-fit	Monopolar	5
Levy et al^36^ (2016)	52	2.5	15	Press-fit	Monopolar	0
Lobo-Escolar et al^37^ (2021)	57	11.7	45	Press-fit	Monopolar	7
Lott et al^38^ (2018)	52	2.8	68	Press-fit	Monopolar	2
Marinelli et al^39^ (2025)	49	14.5	38	Mixed	Mixed	3
Marsh et al^40^ (2016)	48	8.2	55	Loose-fit	Monopolar	0
Mercer et al^41^ (2022)	53	4.1	52	Press-fit	Bipolar	4
Moghaddam et al^42^ (2016)	42	3.5	75	Loose-fit	Monopolar	4
Mukka et al^44^ (2020)	56	6.0	30	Mixed	Mixed	4
Nieboer et al^46^ (2022)	50	2.3	116	Mixed	Mixed	12
Nolte et al^47^ (2021)	53	3.7	123	Mixed	Monopolar	16
Raven et al^49^ (2020)	54	4.0	86	Mixed	Mixed	6
Raven et al^50^ (2023)	53	10.2	20	Loose-fit	Monopolar	0
Rodriguez-Quintana et al^52^ (2017)	49	2.0	28	Mixed	Monopolar	2
Rota et al^53^ (2025)	45	12.5	63	Cemented	Bipolar	9
Rotini et al^54^ (2012)	46	2.0	31	Press-fit	Bipolar	2
Schnetzke et al^55^ (2021)	52	3.1	234	Press-fit	Monopolar	17
Shimura et al^56^ (2025)	63	3.3	32	Mixed	Monopolar	2
Shore et al^57^ (2008)	43	8.0	32	Loose-fit	Monopolar	0
Songy et al^59^ (2021)	48	4.0	114	Mixed	Mixed	14
Strelzow et al^61^ (2017)	50	7.0	148	Press-fit	Monopolar	8
Tarallo et al^62^ (2025)	51	7.0	149	Press-fit	Bipolar	7
Vismara et al^64^ (2025)	59	0.8	44	Press-fit	Bipolar	1
Viswanath et al^65^ (2022)	51	5.8	36	Press-fit	Bipolar	2
Zunkiewicz et al^69^ (2012)	47	2.8	30	Loose-fit	Monopolar	1

Intentional loose-fit (smooth/polished stem) designs were reported in 19 studies (1,611 elbows), press-fit stems in 28 studies (2,856 elbows), and cemented stems in 4 studies (198 elbows); studies reporting outcomes for more than one implant design contributed separate subgroups; no individual elbow was counted twice. Monopolar implants were used in 34 studies (3,612 elbows) and bipolar in 15 studies (855 elbows). Press-fit cohorts had a median follow-up of 6.2 years, whereas intentional loose-fit cohorts had a median follow-up of 3.8 years; this systematic difference in surveillance duration underpins the need for time-adjusted incidence-rate analysis.

Risk of bias assessment using Methodological Index for Non-Randomized Studies showed moderate-to-high risk in 68% of domains, primarily due to retrospective design and lack of prospective calculation of study size. Grading of Recommendations Assessment, Development and Evaluation assessment rated overall quality of evidence as low for revision and reoperation outcomes.

Outcomes stratified by fixation were reported in 60 subgroups involving more than 3,500 elbows. In the unadjusted pooled analysis (Table II, Supplementary Fig. 2), press-fit stems (n = 2,330) demonstrated a revision rate of 8.0% (95% confidence interval [CI]: 5.8-10.5), which was significantly higher than the 3.4% (95% CI: 1.6-5.1) rate observed in intentional loose-fit stems (n = 1,048; *P* < .001). Cemented stems (n = 194) demonstrated the highest unadjusted revision rate at 8.8% (95% CI: 4.6-13.8).

**Table II:** Pooled revision rates (conventional random-effects meta-analysis) following primary metallic radial head arthroplasty, stratified by stem fixation (intentional loose-fit, press-fit, cemented) and implant polarity (monopolar vs. bipolar)

Implant characteristic	Subgroups (n)	Elbows (n)	Pooled revision rate (95% CI)	I^2^ (%)	*P* value
Stem fixation					**<.001**
Loose-fit	19	1,048	3.4% (1.6-5.1)	40.0	
Press-fit	34	2,330	8.0% (5.8-10.5)	65.3	
Cemented	7	194	8.8% (4.6-13.8)	14.1	
Implant polarity					**.74**
Monopolar	37	2,802	5.8% (4.0-7.8)	65.6	
Bipolar	12	309	5.8% (2.0-11.1)	52.1	

The values showed in bold are the *P* values for the tests of subgroup differences.

*CI*, confidence interval.

**Figure 2:**
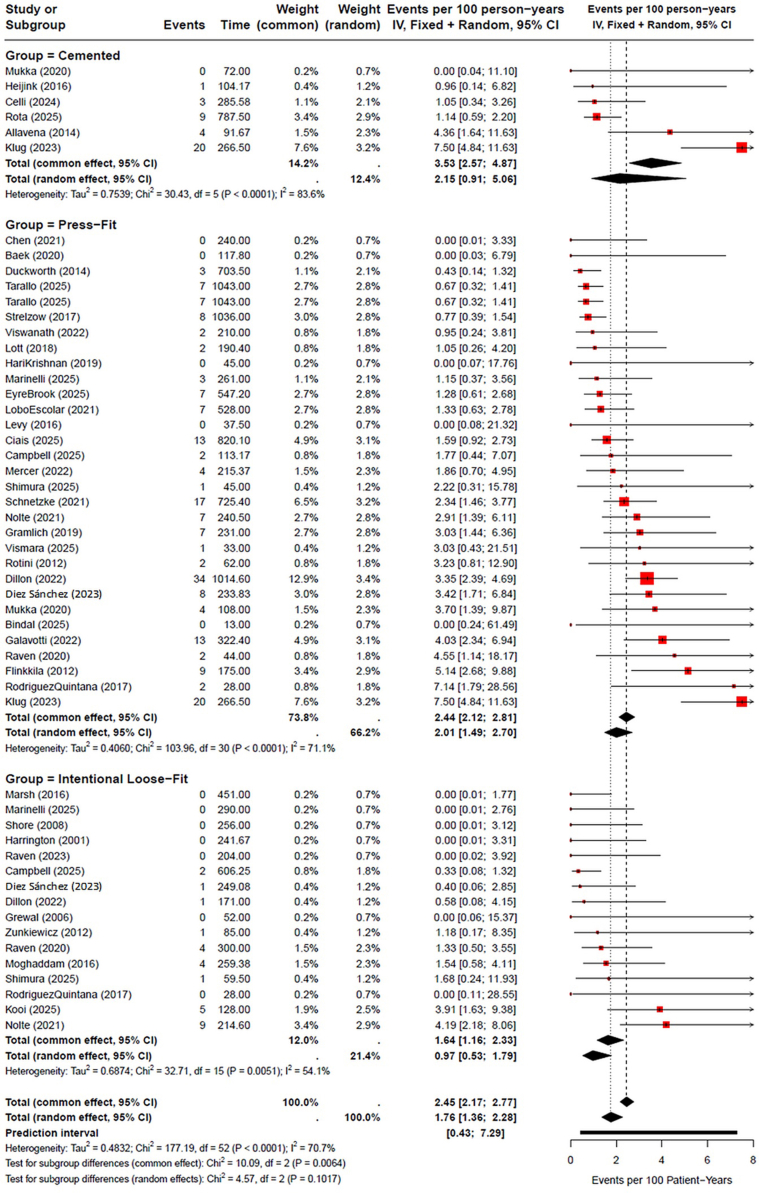
Forest plot of revision incidence rates (events per 100 patient-years, random-effects meta-analysis) following primary metallic radial head arthroplasty, stratified by stem fixation philosophy (intentional loose-fit vs. press-fit vs. cemented). Total patient-years of exposure are displayed for each subgroup.

The statistical difference between fixation strategies was lost when adjusting for the duration of implantation using incidence rate analysis (Table III; Fig. 2). Press-fit stems had an incidence rate of 2.01 events per 100 patient-years (192 events over 10,693 patient-years), compared to 0.97 events per 100 patient-years for loose-fit stems (28 events over 3,596 patient-years). Cemented stems demonstrated a comparable incidence rate of 2.15 events per 100 patient-years (37 events over 1,607 patient-years). The test for subgroup differences was not significant (*P* = .102). Although the incidence-rate point estimate for press-fit stems remained numerically higher (2.01 vs. 0.97), the wide CIs and overlapping uncertainty suggest residual confounding cannot be excluded.

**Table III:** Incidence-rate meta-analysis (revisions per 100 patient-years) following primary metallic radial head arthroplasty, stratified by stem fixation philosophy and implant polarity, with total patient-years exposure shown for each subgroup

Fixation type	Events (n)	Patient-years	Incidence rate (per 100 patient-years)	95% CI	*P* value
Stem fixation					**.102**
Loose-fit	28	3,596	0.97	0.53-1.79	
Press-fit	192	10,693	2.01	1.49-2.70	
Cemented	37	1,607	2.15	0.91-5.06	
Implant polarity					**.666**
Monopolar	188	12,020	1.64	1.18-2.27	
Bipolar	24	1,650	1.89	1.07-3.35	

The values showed in bold are the *P* values for the tests of subgroup differences.

*CI*, confidence interval.

**Figure 3:**
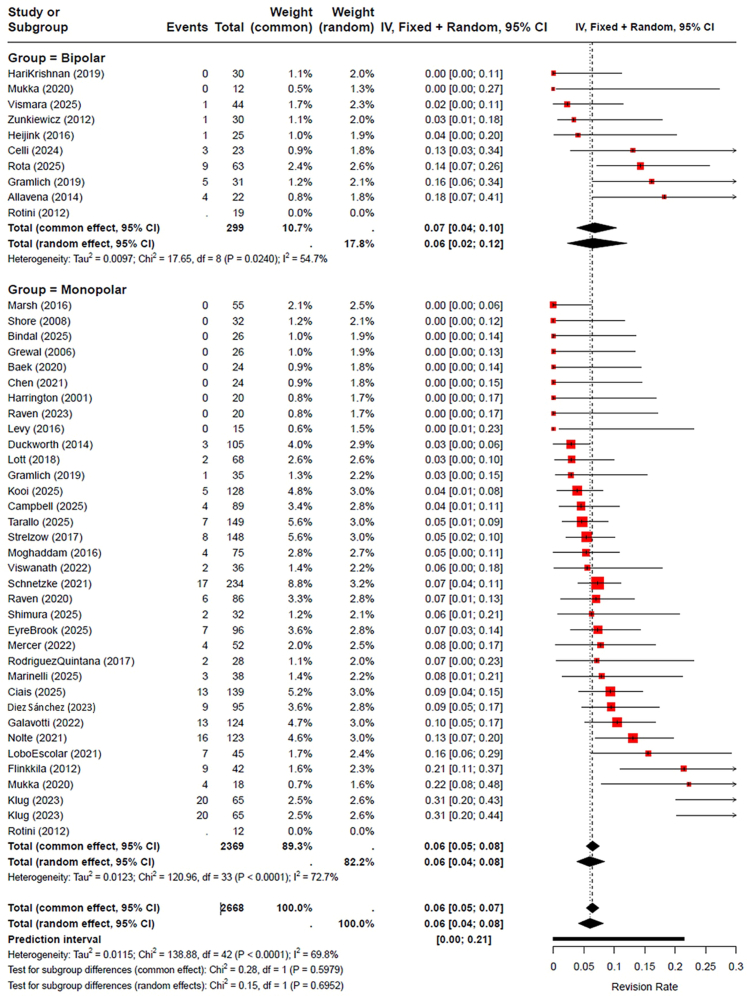
Forest plot of revision incidence rates (events per 100 patient-years, random-effects meta-analysis) following primary metallic radial head arthroplasty, stratified by implant polarity (monopolar vs. bipolar). Total patient-years of exposure are displayed for each subgroup.

There was no difference in outcomes based on implant polarity. Unadjusted revision rates were identical for monopolar (n = 2,802) and bipolar (n = 309) designs (5.8% vs. 5.8%; *P* = .74). Similarly, incidence rate analysis confirmed no difference in survivorship per patient-year (1.64 vs. 1.89; *P* = .666) (Fig. 3).

In the unadjusted pooled proportion analysis of reoperations, not including revisions (Table IV), loose-fit stems (n = 771) demonstrated a reoperation rate of 5.7% compared to 11.5% for press-fit stems (n = 1,593), while cemented stems demonstrated a rate of 20.4% (n = 173). No differences were found (*P* = .054).

**Table IV:** Pooled reoperation rates following primary radial head arthroplasty, stratified by stem fixation philosophy

Outcome	Subgroups (n)	Elbows (n)	Pooled rate (95% CI)	*P* value
Any reoperation				**.054**
Loose-fit	14	771	5.7% (3.1-15.2)	
Press-fit	28	1,593	11.5% (8.5-15.2)	
Cemented	5	173	20.4% (6.4-49.3)	

The values showed in bold are the *P* values for the tests of subgroup differences.

*CI*, confidence interval.

Random-effects meta-regression at the subgroup level (53 subgroups; Supplementary Table 1) explored sources of heterogeneity in the incidence-rate model. The univariable comparison of loose-fit vs. press-fit stems yielded an IRR of 0.55 (95% CI: 0.29-1.01, *P* = .055), directionally consistent with the primary subgroup analysis but not reaching the a priori threshold of 0.01. Cemented stems did not differ significantly from press-fit (IRR: 1.11, 95% CI: 0.51-2.43, *P* = .79). Study-level mean patient age (*P* = .30) and publication year (*P* = .76) were not significant moderators. In contrast, mean follow-up duration was strongly inversely associated with the reported incidence rate: each additional year of mean follow-up was associated with a 14% lower reported revision rate (IRR: 0.86, 95% CI: 0.80-0.92, *P* < .001), and inclusion of this moderator reduced residual I^2^ from 71.3% to 58.5%. This finding empirically supports the bathtub-shaped hazard model and indicates that the constant-hazard assumption underlying incidence-rate meta-analysis is violated, with early failures predominating. In the multivariable model adjusting for mean age and publication year, the loose-fit vs. press-fit comparison was essentially unchanged (IRR: 0.56, 95% CI: 0.29-1.06, *P* = .073).

## Discussion

The principal finding of this systematic review and meta-analysis is that the survival advantage frequently observed with intentional loose-fit stems is significantly attenuated when adjusted for surveillance duration. In our unadjusted pooled analysis, press-fit stems demonstrated a revision rate of 8.0%, significantly higher than the 3.4% rate observed in loose-fit stems (*P* < .001). However, the press-fit cohorts included in this analysis contributed more than 10,000 patient-years of exposure compared to just 3,500 for loose-fit stems. When adjusted for this disparity using incidence-rate meta-analysis (normalizing events per 100 patient-years of exposure), the statistical difference in revision rates was no longer significant (*P* = .102). However, it is critical to note that the point estimate for failure in press-fit stems (2.01) remained double that of loose-fit stems (0.97). While we cannot reject the null hypothesis, the data suggest that the historical superiority of loose-fit stems is exaggerated by surveillance bias, but likely not eliminated entirely. The lack of statistical significance should not be interpreted as definitive proof of equivalence.^4^ While our findings do not prove equivalence, they reduce confidence in the previously assumed superiority of loose-fit stems. Surgeons should recognize that differences in reported failure rates may largely reflect follow-up duration rather than inherent mechanical behavior.^18^^,^^35^ This interpretation is reinforced by our meta-regression, in which neither patient age nor publication year explained the between-study heterogeneity, whereas follow-up duration was strongly associated with the reported revision rate. Taken together, these analyses indicate that the apparent superiority of loose-fit stems is driven largely by differences in follow-up duration between cohorts rather than by implant design.

While the rate of revision (stem exchange or removal) was statistically equivalent after time correction, our analysis of reoperations, not including revision, showed a trend favoring loose-fit designs. Loose-fit stems demonstrated a lower absolute rate of reoperation (5.7%) compared to press-fit stems (11.5%). Although this difference did not reach statistical significance (*P* = .054), it warrants further investigation. Unlike other joint arthroplasties, which are commonly performed by a subspecialist, RHA is often performed by generalists in the context of trauma. Intentional loose-fit stems, acting as smooth spacers that allow toggling, may offer a distinct clinical profile by accommodating minor malalignments. Loose-fit constructs may tolerate imperfect canal preparation by permitting controlled micromotion, whereas press-fit fixation may convert small alignment errors into eccentric loading that accelerates loosening.^10^

Regarding implant polarity, our analysis found no survival benefit associated with bipolar designs. We found identical unadjusted revision rates (5.8%) for both monopolar and bipolar implants (*P* = .74). Crucially, this equivalence persisted after adjusting for surveillance duration, with incidence-rate analysis demonstrating no significant difference between the groups (1.64 vs. 1.89 per 100 patient-years; *P* = .666). Theoretical arguments that bipolar heads improve radio-capitellar tracking have not translated into improved medium-term to long-term survivorship in this dataset. Given that bipolar assemblies introduce modular failure modes, specifically polyethylene wear and dissociation,^66^ and incur higher implant costs, the current evidence does not support a routine survival benefit. Monopolar implants therefore remain a reasonable default in the absence of a demonstrated polarity-related survival advantage, although formal cost-effectiveness analyses and longer-term comparative data are required before this can be regarded as definitive.

This study has limitations inherent to the available literature. The majority of included studies were retrospective case series with moderate methodological quality, with 82% representing Level IV evidence. A further consideration concerns the primary outcome: revision is a clinician-mediated and patient-mediated event rather than a pure marker of mechanical failure, as patients may tolerate suboptimal outcomes to avoid further surgery. Reported revision rates may therefore underestimate true mechanical failure, although the direction and magnitude of any such effect cannot be determined from the available data. Although our incidence-rate analysis mitigates surveillance bias at the study level, it cannot fully replicate individual patient-level survival analysis. Furthermore, the definition of “reoperation” varied between studies, potentially introducing heterogeneity. Third, incidence-rate meta-analysis assumes a constant hazard rate (linear failure risk over time), whereas arthroplasty survival often follows a “bathtub” curve or wear-dependent degradation.^45^ Consequently, this method cannot detect early concentrated failures or model distinct failure phases. If press-fit stems are prone to early aseptic loosening, “smearing” these events over total patient-years may overcorrect for surveillance bias, potentially masking a true difference; individual patient-level time-to-event data would be required for such nuanced modeling (eg, hazard curves, competing-risk analysis). Finally, to ensure granular stratification by fixation type, we excluded large registry datasets that lacked implant-level coding. While this reduced our total sample size compared to registry-based reports, it ensured that failure events were attributed to the correct design philosophy.

## Conclusion

Surveillance time, not implant design, accounts for much of the apparent variation in RHA survivorship. Once exposure time is taken into account, the revision advantage previously attributed to intentional loose-fit stems is no longer evident, and implant polarity shows no association with survivorship. Loose-fit, press-fit, and monopolar designs offer broadly comparable revision-free survival on the current evidence, and the additional cost and complexity of bipolar implants were not associated with improved outcomes. Surgeons should therefore interpret published failure rates in the context of study follow-up and may reasonably base implant selection on cost, availability, and intraoperative factors rather than on presumed differences in survivorship. Confirmation through registry data with patient-level survival analysis remains a priority.

## Disclaimers:

Funding: No funding was disclosed by the authors.

Conflicts of interest: The authors, their immediate families, and any research foundations with which they are affiliated have not received any financial payments or other benefits from any commercial entity related to the subject of this article.

## References

[1] Acevedo D.C., Paxton E.S., Kukelyansky I., Abboud J., Ramsey M. (2014). Radial head arthroplasty: state of the art. J Am Acad Orthop Surg.

[2] Agyeman K.D., Damodar D., Watkins I., Dodds S.D. (2019). Does radial head implant fixation affect functional outcomes? A systematic review and meta-analysis. J Shoulder Elbow Surg.

[3] Allavena C., Delclaux S., Bonnevialle N. (2014). Outcomes of bipolar radial head prosthesis to treat complex radial head fractures in 22 patients with a mean follow-up of 50 months. Orthop Traumatol Surg Res.

[4] Altman D.G., Bland J.M. (1995). Absence of evidence is not evidence of absence. BMJ.

[5] Baek C., Kim B., Kim D. (2020). Short- to mid-term outcomes of radial head replacement for complex radial head fractures. Clin Shoulder Elbow.

[6] Balduzzi S., Rücker G., Schwarzer G. (2019). How to perform a meta-analysis with R: a practical tutorial. Evid Based Ment Health.

[7] Bindal S., Pooni H.S., Garg R. (2025). Functional outcomes of radial head arthroplasty in Mason type III and IV fractures. World J Orthop.

[8] Campbell B.R., Rengifo S., Wickes C.B. (2025). Radial head arthroplasty for fracture: implant survivorship and outcomes at mean follow-up of 8 years. J Hand Surg Am.

[9] Celli A., Paroni C., Bonucci P. (2024). Long-terms outcomes of radial head arthroplasty using a bipolar prosthesis. Arch Orthop Trauma Surg.

[10] Chanlalit C., Shukla D.R., Fitzsimmons J.S., An K., O'Driscoll S.W. (2012). Stress shielding around radial head prostheses. J Hand Surg Am.

[11] Chen A.C., Cheng Y., Chiu C. (2021). Long-term outcomes of radial head arthroplasty in complex elbow fracture dislocation. J Clin Med.

[12] Chytas I.D., Antonopoulos C., Cheva A., Givissis P. (2018). Capitellar erosion after radial head arthroplasty: a comparative biomechanical study of operated radial head fractures on cadaveric specimens. Orthop Traumatol Surg Res.

[13] Ciais G., Tibbo M., Massin V. (2025). Short- to midterm outcomes of 139 pyrocarbon monopolar radial head arthroplasties. J Shoulder Elbow Surg.

[14] Davey M.S., Davey M.G., Hurley E.T., Galbraith J.G., Molony D., Mullett H. (2021). Long-term outcomes of radial head arthroplasty for radial head fractures-a systematic review at minimum 8-year follow-up. J Shoulder Elbow Surg.

[15] Diez Sánchez B., Barco R., Antuña S.A. (2023). Radial head replacement for acute complex elbow instability: a long-term comparative cohort study of 2 implant designs. J Shoulder Elbow Surg.

[16] Dillon M.T., Dontsi M., Alabaster A. (2022). Patient- and procedure-specific variables associated with removal or revision of radial head arthroplasty. Perm J.

[17] Duckworth A.D., Wickramasinghe N.R., Clement N.D. (2014). Radial head replacement for acute complex fractures: what are the rate and risks factors for revision or removal?. Clin Orthop Relat Res.

[18] Evans J.T., Evans J.P., Walker R.W., Blom A.W., Whitehouse M.R., Sayers A. (2019). How long does a hip replacement last? A systematic review and meta-analysis of case series and national registry reports with more than 15 years of follow-up. Lancet.

[19] Eyre-Brook A.I., Kankanalu P., Majkowski L. (2025). Outcomes of press-fit radial head arthroplasty in unconstructable radial head fractures with associated elbow injuries: an average 5-year follow up. Shoulder Elbow.

[20] Flinkkilä T., Kaisto T., Sirniö K. (2012). Short- to mid-term results of metallic press-fit radial head arthroplasty in unstable injuries of the elbow. J Bone Joint Surg Br.

[21] Galavotti C., Padovani S., Nosenzo A. (2022). Radial head arthroplasty: does ligaments repair influence outcomes? A minimum two years follow-up radiographic multi-center study. Eur J Orthop Surg Traumatol.

[22] Gramlich Y., Krausch E., Klug A. (2019). Complications after radial head arthroplasty: a comparison between short-stemmed bipolar and monopolar long-stemmed osteointegrative rigidly fixed prostheses. Int Orthop.

[23] Grewal R., MacDermid J.C., Faber K.J. (2006). Comminuted radial head fractures treated with a modular metallic radial head arthroplasty. Study of outcomes. J Bone Joint Surg Am.

[24] Guyatt G.H., Oxman A.D., Vist G.E., Kunz R., Falck-Ytter Y., Alonso-Coello P. (2008). GRADE: an emerging consensus on rating quality of evidence and strength of recommendations. BMJ.

[25] Hari Krishnan B., Gupta T.P. (2019). Bipolar radial head arthroplasty for management of radial head fractures. J Arthrosc Joint Surg.

[26] Harrington I.J., Sekyi-Otu A., Barrington T.W., Evans D.C., Tuli V. (2001). The functional outcome with metallic radial head implants in the treatment of unstable elbow fractures: a long-term review. J Trauma.

[27] Heijink A., Kodde I.F., Mulder P.G.H. (2016). Cemented bipolar radial head arthroplasty: midterm follow-up results. J Shoulder Elbow Surg.

[28] Heijink A., Kodde I.F., Mulder P.G.H., Veltman E.S., Kaas L., van den Bekerom M.P.J. (2016). Radial head arthroplasty: a systematic review. JBJS Rev.

[29] Higgins J.P.T., Thompson S.G., Deeks J.J., Altman D.G. (2003). Measuring inconsistency in meta-analyses. BMJ.

[30] Judet T., Garreau de Loubresse C., Piriou P., Charnley G. (1996). A floating prosthesis for radial-head fractures. J Bone Joint Surg Br.

[31] Kachooei A.R., Claessen F.M.A.P., Chase S.M. (2016). Factors associated with removal of a radial head prosthesis placed for acute trauma. Injury.

[32] Kachooei A.R., Baradaran A., Ebrahimzadeh M.H., van Dijk C.N., Chen N. (2018). The rate of radial head prosthesis removal or revision: a systematic review and meta-analysis. J Hand Surg Am.

[33] Klug A., Jakobi T., Schnetz M. (2023). Mid-term outcome following radial head arthroplasty in acute trauma: risk factors for poor outcome. J Shoulder Elbow Surg.

[34] Kooi K., Stam M., Legerstee I. (2025). Comparing radial head fracture surgical outcomes in patients younger and older than forty. J Orthop Trauma.

[35] Labek G., Thaler M., Janda W., Agreiter M., Stöckl B. (2011). Revision rates after total joint replacement: cumulative results from worldwide joint register datasets. J Bone Joint Surg Br.

[36] Levy J.C., Formaini N.T., Kurowicki J. (2016). Outcomes and radiographic findings of anatomic press-fit radial head arthroplasty. J Shoulder Elbow Surg.

[37] Lobo-Escolar L., Abellán-Miralles C., Escolà-Benet A. (2021). Outcomes of press-fit radial head arthroplasty following complex radial head fractures. Orthop Traumatol Surg Res.

[38] Lott A., Broder K., Goch A. (2018). Results after radial head arthroplasty in unstable fractures. J Shoulder Elbow Surg.

[39] Marinelli A., Riva M., Sessa A. (2025). What happens to the elbow 15 years after a radial head prosthesis? A clinical and imaging long-term follow-up study. J Shoulder Elbow Surg.

[40] Marsh J.P., Grewal R., Faber K.J. (2016). Radial head fractures treated with modular metallic radial head replacement: outcomes at a mean follow-up of eight years. J Bone Joint Surg Am.

[41] Mercer D.M., Bolano L.E., Rubio F. (2022). A radial head prosthesis that aligns with the forearm axis of rotation: a retrospective multicenter study. Semin Arthroplasty JSES.

[42] Moghaddam A., Raven T.F., Dremel E. (2016). Outcome of radial head arthroplasty in comminuted radial head fractures: short and midterm results. Trauma Mon.

[43] Moon J., Berglund L.J., Zachary D., An K., O'Driscoll S.W. (2009). Radiocapitellar joint stability with bipolar versus monopolar radial head prostheses. J Shoulder Elbow Surg.

[44] Mukka S., Sjöholm P., Perisynakis N. (2020). Radial head arthroplasty for radial head fractures: a clinical and radiological comparison of monopolar and bipolar radial head arthroplasty at a mean follow-up of 6 years. Eur J Trauma Emerg Surg.

[45] Murray D.W., Carr A.J., Bulstrode C. (1993). Survival analysis of joint replacements. J Bone Joint Surg Br.

[46] Nieboer M.J., Austin D.C., Uvodich M.E. (2022). Acute versus delayed radial head arthroplasty for the treatment of radial head fractures. J Shoulder Elbow Surg.

[47] Nolte P., Tross A., Groetzner-Schmidt C. (2021). Risk factors for revision surgery following radial head arthroplasty without cement for unreconstructible radial head fractures: minimum 3-year follow-up. J Bone Joint Surg Am.

[48] Page M.J., McKenzie J.E., Bossuyt P.M., Boutron I., Hoffmann T.C., Mulrow C.D. (2021). The PRISMA 2020 statement: an updated guideline for reporting systematic reviews. BMJ.

[49] Raven T.F., Banken L., Schmidmaier G. (2020). Evaluation of two different types of radial head prosthesis in practical use. Using either Evolve or MoPyC radial head prosthesis in the treatment of comminuted radial head fractures. Orthop Rev (Pavia).

[50] Raven T.F., Moghaddam A., Studier-Fischer S. (2023). Clinical long-term results of radial head arthroplasty in comminuted radial head fractures. Musculoskelet Surg.

[51] Ring D., Quintero J., Jupiter J.B. (2002). Open reduction and internal fixation of fractures of the radial head. J Bone Joint Surg Am.

[52] Rodriguez-Quintana D., Comulada D.B., Rodriguez-Quintana N. (2017). Radial head ingrowth anatomic implant versus smooth stem monoblock implant in acute terrible triad injury: a prospective comparative study. J Orthop Trauma.

[53] Rota C., Celli A., Dutto E. (2025). Long-term outcomes of a cemented bipolar radial head prosthesis: a large retrospective study. J Shoulder Elbow Surg.

[54] Rotini R., Marinelli A., Guerra E. (2012). Radial head replacement with unipolar and bipolar SBi system: a clinical and radiographic analysis after a 2-year mean follow-up. Musculoskelet Surg.

[55] Schnetzke M., Jung M.K., Groetzner-Schmidt C. (2021). Long-term outcome and survival rate of monopolar radial head replacement. J Shoulder Elbow Surg.

[56] Shimura H., Wakabayashi Y., Yamada T. (2025). Radiographic and clinical comparisons between loose-fit and press-fit stems in monopolar radial head arthroplasty for comminuted radial head fractures. J Orthop Sci.

[57] Shore B.J., Mozzon J.B., MacDermid J.C. (2008). Chronic posttraumatic elbow disorders treated with metallic radial head arthroplasty. J Bone Joint Surg Am.

[58] Slim K., Nini E., Forestier D., Kwiatkowski F., Panis Y., Chipponi J. (2003). Methodological index for non-randomized studies (minors): development and validation of a new instrument. ANZ J Surg.

[59] Songy C.E., Kennon J.C., Barlow J.D. (2021). Radial head replacement for acute radial head fractures: outcome and survival of three implant designs with and without cement fixation. J Orthop Trauma.

[60] Stijnen T., Hamza T.H., Ozdemir P. (2010). Random effects meta-analysis of event outcome in the framework of the generalized linear mixed model with applications in sparse data. Stat Med.

[61] Strelzow J.A., Athwal G.S., MacDermid J.C. (2017). Effect of concomitant elbow injuries on the outcomes of radial head arthroplasty: a cohort comparison. J Orthop Trauma.

[62] Tarallo L., Celli A., Benedetti L. (2025). Long-term survival of Acumed anatomical radial head implant for Mason type III-IV fractures: a 15-year follow-up. J Shoulder Elbow Surg.

[63] Thyagarajan D.S. (2022). Radial head replacement – a comprehensive review. J Orthop.

[64] Vismara V., Guerra E., Accetta R. (2025). Radial head prosthesis with interconnected porosity showing low bone resorption around the stem. J Clin Med.

[65] Viswanath A.I., Watts A.C. (2022). Survivorship of anatomic press-fit short-stem radial head replacement with a pyrocarbon bearing. Shoulder Elbow.

[66] van Riet R.P., Sanchez-Sotelo J., Morrey B.F. (2010). Failure of metal radial head replacement. J Bone Joint Surg Br.

[67] Vannabouathong C., Venugopal N., Athwal G.S., Moro J., Bhandari M. (2020). Radial head arthroplasty: fixed-stem implants are not all equal-a systematic review and meta-analysis. JSES Int.

[68] Wan X., Wang W., Liu J., Tong T. (2014). Estimating the sample mean and standard deviation from the sample size, median, range and/or interquartile range. BMC Med Res Methodol.

[69] Zunkiewicz M.R., Clemente J.S., Miller M.C. (2012). Radial head replacement with a bipolar system: a minimum 2-year follow-up. J Shoulder Elbow Surg.

