# Supplementary figures and images for: Stem design in radial head arthroplasty: a systematic review and meta-analysis
Source: J Shoulder Elb Arthroplast. 2026 Jun 26;10(3):100052. doi: 10.1016/j.jsea.2026.100052 (PMC13392940; doi:10.1016/j.jsea.2026.100052)

**Supplementary Figure 1 - Search Strategy**

**
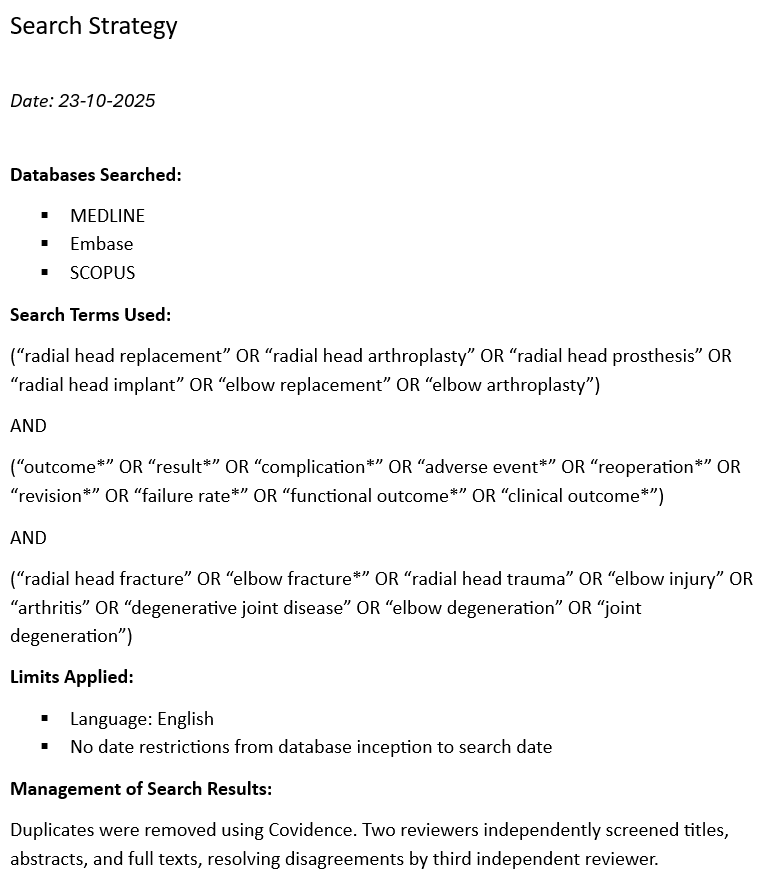
**

Supplement: Supplementary Figure S1 [file mmc1.docx]
