# Supplementary material for: Stem design in radial head arthroplasty: a systematic review and meta-analysis
Source: J Shoulder Elb Arthroplast. 2026 Jun 26;10(3):100052. doi: 10.1016/j.jsea.2026.100052 (PMC13392940; doi:10.1016/j.jsea.2026.100052)

**Supplementary Figure 2 - Forest plot showing pooled unadjusted revision rates (conventional random-effects meta-analysis with logit transformation) following primary metallic radial head arthroplasty, stratified by stem fixation philosophy.**


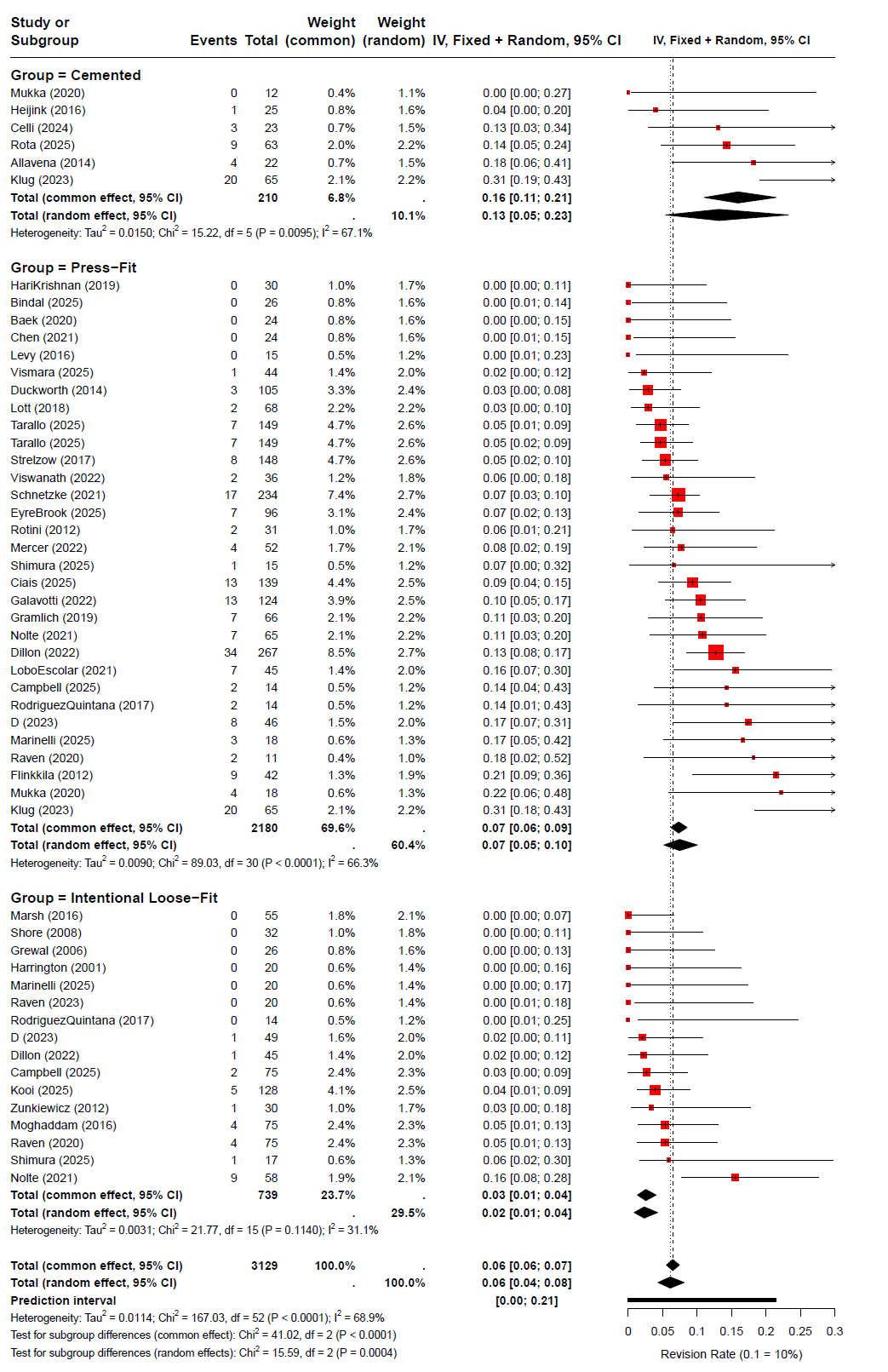

Supplement: Supplementary Figure S2 [file mmc2.docx]
