# Supplementary material for: Stem design in radial head arthroplasty: a systematic review and meta-analysis
Source: J Shoulder Elb Arthroplast. 2026 Jun 26;10(3):100052. doi: 10.1016/j.jsea.2026.100052 (PMC13392940; doi:10.1016/j.jsea.2026.100052)
Supplement: Supplementary Table S1 [file mmc3.docx]

**Supplementary Table 1 — Random-effects meta-regression of revision incidence rate following primary radial head arthroplasty**

*Effect estimates are presented as incidence-rate ratios (IRR) with 95% confidence intervals. The outcome is the log incidence rate of revision per patient-year (subgroup level, n = 53 subgroups). Between-study variance (τ²) estimated using DerSimonian–Laird. Continuity correction of 0.5 applied to zero-event subgroups.*

| **Model / Moderator** | **k** | **IRR (95% CI)** | **p** | **τ²** | **Residual I² (%)** |
| --- | --- | --- | --- | --- | --- |
| **Univariable: Fixation (ref = press-fit)** | 53 | — | — | 0.49 | 71.0 |
| Loose-fit vs press-fit |  | 0.55 (0.29–1.01) | 0.055 |  |  |
| Cemented vs press-fit |  | 1.11 (0.51–2.43) | 0.79 |  |  |
| **Univariable: Mean age (per 10-year increase)** | 53 | 1.38 (0.75–2.53) | 0.30 | 0.50 | 71.9 |
| **Univariable: Publication year (per 5-year increase)** | 53 | 1.04 (0.79–1.39) | 0.76 | 0.49 | 71.9 |
| **Univariable: Mean follow-up (per additional year)** | 53 | **0.86 (0.80–0.92)** | **< 0.001** | 0.27 | 58.5 |
| **Multivariable: Fixation + mean age + publication year** | 53 | — | — | 0.52 | 71.9 |
| Loose-fit vs press-fit |  | 0.56 (0.29–1.06) | 0.073 |  |  |
| Cemented vs press-fit |  | 1.15 (0.52–2.57) | 0.73 |  |  |
| Mean age (per year) |  | 1.03 (0.96–1.10) | 0.38 |  |  |
| Publication year (per year) |  | 0.99 (0.93–1.05) | 0.75 |  |  |

*k = number of subgroups contributing to the model. IRR = incidence-rate ratio. Bold IRR/p indicates statistical significance at α = 0.01 (pre-specified threshold for the primary analysis).*
